# Commentary: Dysregulated Microbial Fermentation of Soluble Fiber Induces Cholestatic Liver Cancer

**DOI:** 10.3389/fcimb.2019.00155

**Published:** 2019-05-21

**Authors:** Baolei Jia, Ruiming Wang, Jie Zhang, Yuxia Chi

**Affiliations:** State Key Laboratory of Biobased Material and Green Papermaking, School of Bioengineering, Qilu University of Technology (Shandong Academy of Sciences), Jinan, China

**Keywords:** gut microbiome, soluble fiber, liver cancer, bile acids, immune regulation

In this article, Singh et al. have shown that feeding innate immune-deficient mice a diet enriched in soluble fibers (including inulin, pectin, and fructo-oligosaccharides) but not insoluble fibers could induce liver cancer (Figure 1) (Singh et al., 2018). Fiber-induced liver cancer did not occur in WT mice, however, WT mice fed a high-fat diet (HFD) could be susceptible to liver cancer upon consumption of soluble fibers. These induced cancers are initiated with cholestasis, followed by hepatocyte death and neutrophilic inflammation in the liver. Liver cancer is influenced by diet and dependent on microbiota; a previous dysbiosis should be required for carcinoma development. The mice model that developed liver cancer in this study displayed a dysbiosis characterized by accumulation of fiber-fermenting bacteria and proteobacteria. Cohousing or cross-fostering of dysbiotic mice with WT mice indicated that liver cancer is transmissible, which further verified a causative role of microbiota in fiber-induced liver cancer.

**Figure 1:**
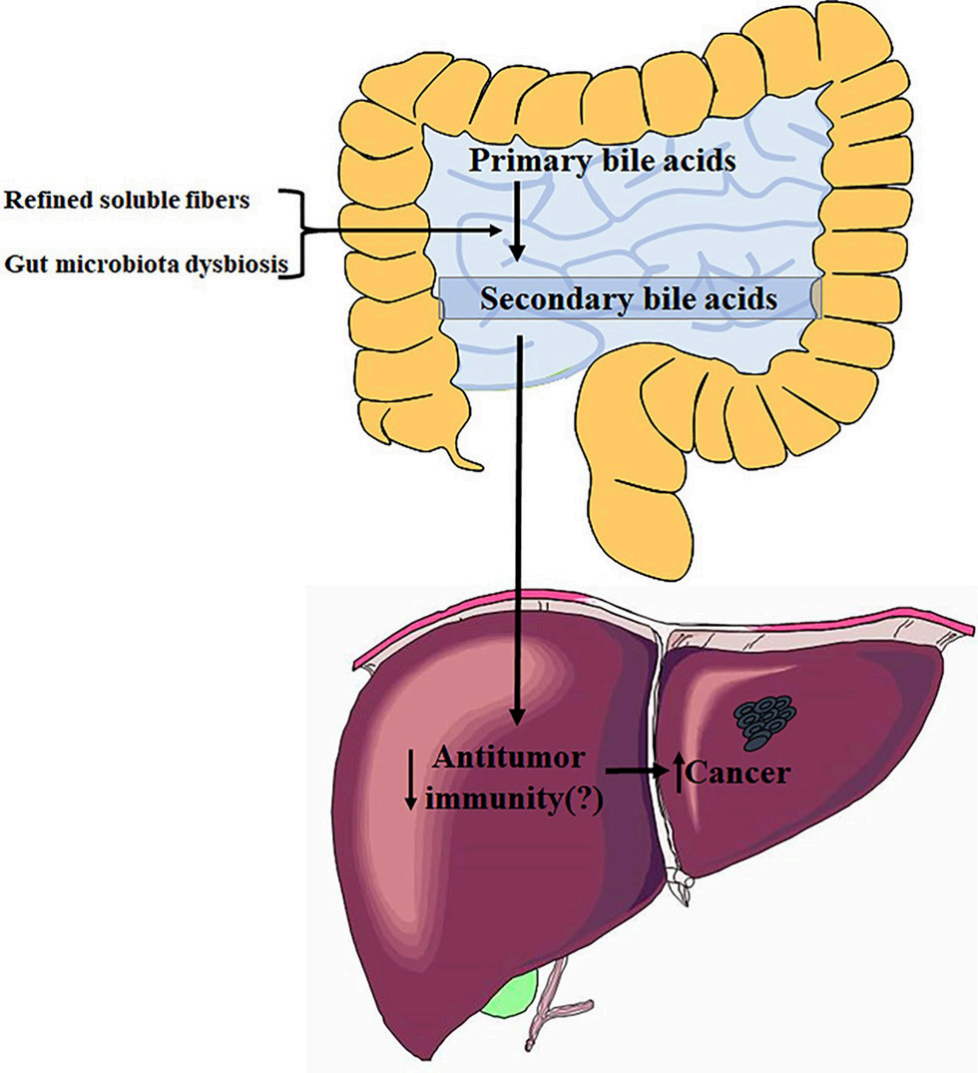
Influence of refined soluble fibers on gut microbiota dysbiosis and liver health. The figure summarizes data from Singh et al. (2018).

Soluble fibers that can be fermented by gut microbiota include inulin, resistant starch, resistant maltodextrins, soluble corn fiber, and polydextrose. Such soluble fibers are recognized as prebiotics because they can be fermented to short-chain fatty acids (SCFAs), which are important for human health (Holscher, 2017). High inulin intake (20%) has been shown to reduce microbiota encroachment, restore lost enterocyte proliferation due to HFD, and improve gut health in mice (Zou et al., 2018). The authors of the current article initially tested whether inulin could alleviate metabolic syndrome in TLR5 knockout (*T5*KO) mice, by feeding the mice an inulin-containing diet (ICD). They observed that the sera of ~40% of mice without metabolic syndrome showed a yellow color, caused by elevated bilirubin levels during the experiments. Finally, based on their observations, the authors concluded that refined fermentable fibers can induce cholestasis followed by liver cancer in mice (Singh et al., 2018). Another study showed that inulin-type fructans have anti-tumorigenic effects in colon cancer (Pool-Zobel, 2007). The contrasting conclusion of the current study extends our knowledge of the effects of various types of dietary fiber on health. The findings support the idea that whether soluble fibers and their fermentation products are beneficial is highly dependent on internal and environmental conditions (Perry et al., 2016). The study also indicated that enrichment of foods with dietary fibers to provide health benefits should be practiced with great caution.

The authors of this study showed that Clostridia species are enriched in the guts of mice with live cancer, including the *Clostridium* cluster XIVa, which performs a key enzymatic transformation of primary to secondary bile acids in the gut (Ridlon et al., 2014). Previous studies have indicated that *Clostridium* cluster XIVa and the secondary bile acids produced by them play important roles in promoting obesity-associated liver cancer (Figure 1). The accumulation of secondary bile acids in the livers of HFD mice suppresses anti-tumor immunity through a PTGER4 receptor on CD8 cells, which is mediated by the Toll-like receptor 2 (TLR2) signaling pathway, thereby contributing to liver cancer progression (Yoshimoto et al., 2013; Loo et al., 2017). Bile acids can also function as messengers to regulate liver cancer by controlling the accumulation of hepatic natural killer T (NKT) cells in a chemokine-dependent manner (Ma et al., 2018). The current research advances these studies by showing that gut microbiota can induce liver cancer in inulin-fed *T5*KO mice, and that oncogenic bacteria are transmissible to susceptible mice, as demonstrated by co-housing or cross-fostering. The article did not identify specific oncogenic bacteria. Overall, these studies on relationships between gut microbiota and liver cancer suggest that *Clostridium* cluster XIVa, *Clostridium* cluster XI, or other strains catalyzing the transformation from primary to secondary bile acids could be promoters of liver oncogenesis.

## Author Contributions

BJ wrote the commentary. RW, JZ, and YC helped to write.

### Conflict of Interest Statement

The authors declare that the research was conducted in the absence of any commercial or financial relationships that could be construed as a potential conflict of interest.
